# A Multimodal Feature Sensing and Fusion Neural Network for Damage Localization by Ultrasonic Guided Waves

**DOI:** 10.3390/s26144525

**Published:** 2026-07-16

**Authors:** Lin Zhang, Lin Mei, Yuxin Bai, Yu Zeng, Zhiqiang Duan, Sida Chen, Qingying Li, Jing Peng, Shuaiyong Li

**Affiliations:** 1 Chongqing Special Equipment Inspection and Research Institute, Chongqing 401121, China; zlydyxf02@163.com (L.Z.); justcoffee123@163.com (S.C.); liqingying@cqtj.org (Q.L.); 2 Key Laboratory of Electromechanical Equipment Security in Westem Complex Environment, State Administration for Market Regulation, Chongqing 401121, China; 3 Chongqing Gas Group Co., Ltd., Chongqing 400025, China; baiyuxin5277@gmail.com; 4 Key Laboratory of Industrial Internet of Things and Networked Control, Ministry of Education, Chongqing University of Posts and Telecommunications, Chongqing 400065, China

**Keywords:** neural network, multimodal feature, damage localization, ultrasonic guided wave (UGW)

## Abstract

Damage localization based on ultrasonic guided waves (UGWs) ensures the reliability and safety of composites. Efficient and accurate damage localization requires full integration of different modality features. However, existing deep learning-based damage localization methods usually focus on single-modal features and cannot deeply mine and fuse different features. In this paper, we propose a novel Multimodal Feature Sensing and Fusion Neural Network (MSFN) for damage localization by UGWs in composites. This method uses an innovative multimodal input mode, in which three different modal signals, namely, the damage signal, scattered wave signal, and energy density signal, are fed into the network as inputs. We use Convolutional Neural Networks (CNNs), Gated Recurrent Units (GRUs) and Bidirectional Gated Recurrent Units (BiGRUs) to construct specific encoders for the characteristics of the three signals to extract the features of different modalities efficiently and quickly. Then we employ an attention mechanism-guided feature fusion strategy to aggregate the various features, map out the correlation between the damage zones and the signal features, and finally decode them through successive linear layers to output the final damage localization results. Subsequent experimental results show that the damage localization accuracy of the MSFN can reach 98.13% even under noise interference. It is shown that its robustness and accuracy are much better than those of other existing networks and it has better localization speed and generalization. The proposed MSFN architecture comprises a CNN-based DS-encoder, a GRU-based SW-encoder, and a BiGRU-based ES-encoder, followed by an attention-guided fusion module, demonstrating its feasibility for near-real-time SHM applications.

## 1. Introduction

Carbon fiber-reinforced polymer (CFRP) composites are extensively utilized in various complex engineering applications due to their advantageous properties, such as light weight, high strength, and fatigue resistance [1]. In the aerospace sector, the properties of these materials can significantly reduce fuel consumption and enable spacecraft to carry additional fuel or cargo [2]. However, when exposed to external environmental erosion and forces, composite materials may suffer from various kinds of latent damage, including cracking, delamination, and internal fractures, leading to functional degradation [3,4]. Such damage poses risks of property loss and even serious casualties. Hence, there is an urgent demand for a method that can perform structural health monitoring (SHM) accurately and conveniently without compromising the integrity of the material.

In this context, damage localization based on UGWs presents a promising solution within the existing field of SHM [5,6]. Low-frequency ultrasonic guided waves (20–120 kHz) exhibit excellent characteristics, including long propagation distances, low costs, and the ability to traverse complex structures. They are widely employed to assess the structural health of elongated components such as pipelines, panels, tracks, and cables [7]. For effective damage localization using UGWs, it is essential to establish a sensor network. In this setup, an actuator introduces the guided waves into the composite medium, while the generated signals are captured by receivers. Given that UGWs are highly sensitive to variations in the material structure along the propagation path, the presence of damage can lead to significant alterations in UGW propagation characteristics. Consequently, many studies have employed baseline subtraction methods to extract high-dimensional scattered wave signals, followed by manual feature extraction [8]. Alternatively, damage indicators such as correlation coefficients [9,10] or energy ratios [11] have been analyzed to identify the overall differences between newly recorded signals and baseline signals. However, these methods often rely solely on the variations in a specific damage indicator to determine damage locations, which may result in incomplete characterization of the damage information contained within the signals. The fundamental limitation of conventional time–frequency localization pipelines, such as short-time Fourier transform, spectrogram-based methods, or wavelet thresholding, lies in the inherent trade-off between time and frequency resolution imposed by the Heisenberg uncertainty principle. More critically, micro-crack echoes, which are the primary indicators of early-stage damage in CFRP composites, typically exhibit extremely weak amplitudes, often less than 5% of the baseline signal, and are easily masked by stronger boundary reflections and material scattering. In addition, the dispersive nature of Lamb waves causes different frequency components to propagate at different velocities, leading to signal spreading that further obscures the identification of subtle damage-induced features. Manual thresholding or peak-picking in such noisy and overlapping environments becomes unreliable, especially when the signal-to-noise ratio drops below 10 dB. As a result, these conventional approaches are inadequate for robust micro-damage detection, which calls for data-driven deep learning methods that can automatically learn discriminative, multi-scale feature representations without relying on hand-crafted parameters. With the remarkable success of deep learning, an increasing number of deep learning-based damage localization methods have been implemented in the field of SHM [12]. These methods circumvent the need for manual feature extraction and adaptively establish nonlinear higher-order mapping relationships between damage locations and input signals [13]. The model utilizes these learned mapping relationships to predict the outcomes of previously unlabeled inputs, thereby facilitating the prediction of damage locations in composite materials. Due to the advantages of convolutional operations in feature extraction, many researchers have employed Convolutional Neural Networks (CNNs) for this purpose. For instance, Zhang et al. [14] introduced a time-varying DI feature that preserves temporal information and utilized a 1D-CNN to construct a mapping relationship between this feature and the damage location. Yun et al. [15] implemented a feature extraction technique leveraging residual connections within CNNs, further enhancing localization accuracy. Su et al. [16] proposed a method for the simultaneous localization and quantification of damage in composite plates using Lamb waves in conjunction with a CNN. Wu et al. [17] combined deep Convolutional Neural Networks with continuous wavelet transforms to propose a novel damage detection method. This technique converts sequence signals into time–frequency images through continuous wavelet transforms and employs a CNN to predict and classify these images. Notably, all the aforementioned methods focus on extracting features from single-modal signals. It is also worth noting that recent advances in computer vision have yielded powerful architectures capable of capturing long-range structural dependencies. Representative examples include visual-based structural tracking models and Spatial Transformer Networks [18]. These models are built to explicitly learn spatial transformations and capture global contextual relationships within image data, which enables strong performance on tasks such as object detection and scene understanding. Within the field of structural health monitoring, researchers have investigated vision-driven approaches to detect surface cracks and measure structural displacements from camera imagery. Directly adapting these vision-oriented models to one-dimensional ultrasonic guided wave signals poses considerable challenges. Ultrasonic guided wave datasets take the form of time-series readings governed by unique physical characteristics including wave propagation, dispersion, and attenuation, which sets them apart from spatial image data. Furthermore, the MSFN model proposed in this work targets a separate research objective: the localization of internal and subsurface damage via distributed sensor networks. This research objective differs fundamentally from surface inspection workflows supported by vision algorithms. Although Spatial Transformer Networks and vision-based tracking systems constitute a valuable parallel research branch, these tools cannot be directly deployed within the ultrasonic guided wave damage localization framework presented here. For this reason, the present study excludes such models from its benchmark comparisons. Nevertheless, models fed only with single-modal feature inputs often fail to capture comprehensive damage-related information. This limitation produces inaccurate prediction outputs and weakens overall model robustness. For this reason, research into the design and deployment of multimodal input schemes has attracted growing scholarly attention. Wang et al. [13] proposed a graph-in-graph convolutional network, which constructs spatiotemporal feature representations of guided wave signals and interconnects them into a global graph. This global graph is directly linked to the damage locations, thereby facilitating the prediction of damage locations. Zhuang et al. [19] utilized a multimodal Gated Recurrent Unit Neural Network (MGNN) model for damage detection and localization in CFRP composites. This method employs continuous wavelet transforms to integrate the time–frequency domain features of the input signals, performs feature extraction using GRUs, and ultimately decodes the damage results through a fully connected layer. Although the above method extracts the features of different modal signals, it does not effectively extract the features for each modality. Moreover, the input modalities of the signal are not comprehensive enough. In the subsequent feature fusion process, the above method only adopts the common fusion method of feature map splicing, neglecting the varying capacities of different features to represent damage. Thus, current deep learning methods based on multimodal features still face challenges. Leveraging the adaptive weight assignment advantages of the attention mechanism, Guo et al. [20] employed a channel attention mechanism in the fault diagnosis of bearings, assigning different weights to signal segments at varying times, thereby enhancing the learning capacity for fault-related features. Similarly, Zhang et al. [14] introduced effective squeeze–excitation as a channel attention module, which significantly improved the representational capability of the network.

Building on these advancements, this study proposes an ultrasonic guided wave damage localization method based on a Multimodal Feature Sensing and Fusion Neural Network (MSFN). This method not only incorporates a novel multimodal input mode that ensures the diversity and comprehensiveness of input signals but also features the specific encoders capable of efficiently and effectively extracting targeted features from various modal input signals. In the subsequent feature fusion phase, we adopt an attention mechanism-guided feature fusion strategy for effective feature integration, which enhances the accuracy of damage localization and boosts the network’s robustness and generalization capabilities.

The contributions of this study are summarized as follows:

1. The proposed MSFN is capable of aggregating time-domain and energy information, combining the learning of features characterizing different modality information to further improve the damage localization capability, and the multimodal input mode strengthens the robustness of the network.
2. We design specific encoders for different signals. They can extract the damage features of the signal more efficiently and quickly based on different modality signal characteristics, which improves the accuracy of damage localization in the network.
3. We introduce an innovative feature fusion strategy guided by an attention mechanism, which produces a feature map enriched with global damage feature information. It highlights the relationships between various features and the overarching damage information, thereby enhancing the network’s capacity to accurately characterize the locations of damage.

The remainder of the paper is organized as follows: Section 2 describes the proposed method; Section 3 describes the specific setup details of the experiment; Section 4 illustrates the results and conclusions of the experiment; and in Section 5, we summarize the methodology of the paper and discuss its advantages and shortcomings.

## 2. Materials and Methods

**Figure 1:**
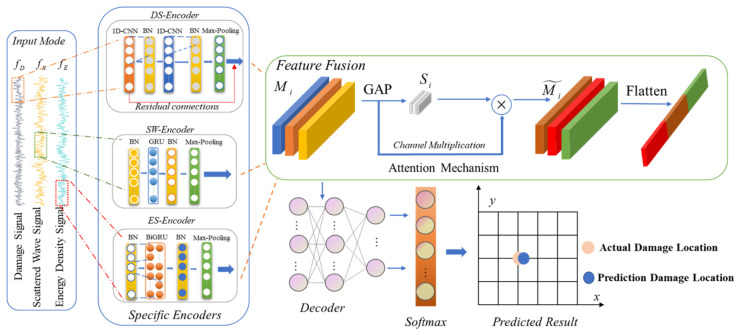
Flowchart of the method based on ultrasonic guided wave damage localization using the MSFN.

The MSFN-based ultrasonic guided wave damage localization method incorporates a multimodal input mode that integrates information from different modalities, specific encoders tailored for various signals, and a feature fusion strategy guided by an attention mechanism. The workflow of the proposed method is illustrated in Figure 1. As depicted in the figure, the method consists of the following four steps:

First, damage signal data collected from multiple sensors undergo denoising and continuous wavelet transformation. It should be noted that the raw 1D time-domain signals collected from the sensors are transformed into 2D time–frequency scalograms via CWT before being fed into the encoders. Second, any set of signals $\left(f_{D_{i}},f_{S_{i}},f_{E_{i}}\right)$ representing different modalities is processed through specialized encoders to generate distinct high-dimensional features $\left(\tilde{f_{D_{i}}},\tilde{f_{S_{i}}},\tilde{f_{E_{i}}}\right)$. The specific encoders comprise three parallel encoders: a damage signal encoder (DS-encoder), a scattered wave signal encoder (SW-encoder), and an energy density signal encoder (ES-encoder). In the subsequent step, we implement an attention mechanism-guided feature fusion strategy. This involves utilizing the weight allocation capabilities of the attention mechanism to efficiently identify and prioritize the critical components of the fused features that signify damage locations. We assign greater weights to features with discriminative properties to ensure that the feature maps provide a more comprehensive representation of damage information. Finally, the final prediction results are obtained through three consecutive linear layers, with the output generated by the softmax layer.

### 2.1. Preprocessing of Signals

The limited number of damage features contained in the unimodal signal results in a poor representation of the network with respect to the damage location. To solve this problem, we use a new multimodal input mode, which combines time-domain features and energy information to make the input features of the network more diverse and comprehensive. The input signal consists of the filtered damage signal, the scattered wave signal obtained from the baseline comparison, and the energy density signal. To clarify, the three input modalities are defined as follows: (1) $f_{D}$ denotes the raw filtered damage signal (1D time-domain voltage sequence); (2) $f_{s}$ denotes the scattered wave signal obtained by subtracting the baseline signal (acquired from the pristine plate) from the damage signal, i.e., $f_{s}$ = $f_{D}$ − $f_{baseline}$; (3) $f_{E}$ denotes the energy density signal, computed as the squared modulus of the CWT coefficients of $f_{s}$. These three modalities represent complementary physical characteristics: the time-domain waveform, wavefield perturbation, and time–frequency energy distribution, respectively. The filtering process employs a fourth-order Butterworth high-pass filter with a cutoff frequency of 20 kHz and a filter order of $n_{F}=3$. It is worth mentioning that because the continuous wavelet transform (CWT) provides information about the damage features associated with the signal in the frequency domain, the localized energy time-domain distribution at instantaneous frequencies can be obtained [19]. The complex Morlet wavelet is chosen because it offers an optimal trade-off between time and frequency localization for dispersive Lamb wave signals. In our preliminary trials, the Daubechies-4 wavelet yielded blurrier scalograms for higher frequency components, reducing localization accuracy by approximately 3–5%. The complex Morlet’s Gaussian-modulated sinusoidal shape also matches the typical ring-down characteristics of UGW bursts, as also observed in 18. Therefore, in this study, the scattered wave signal is processed using CWT to obtain the energy density signal. Equation (1) defines the CWT.

(1) $$C(a,b)=\int_{-\infty }^{+\infty }f(t)\psi^{*}\left(\frac{t-b}{a}\right)dt$$

where C(a,b) denotes the continuous wavelet coefficients, f(t) is the original time domain signal, and $\psi^{*}(t)$ denotes the complex conjugate of the mother wavelet function. a denotes the scale parameter, and b denotes the translation parameter. Due to the smoothness and symmetry of complex Morlet wavelets, they are well-suited for analyzing non-stationary signals. Consequently, in this method, complex Morlet wavelets are selected as the mother wavelet function to enhance analytical precision, and they are shown as follows:

(2) $$\psi (t)=\frac{1}{\sqrt{\pi f_{b}}}e^{\frac{-t^{2}}{f_{b}}}e^{2\pi f_{c}jt}$$

The frequency bandwidth is denoted by $f_{b}$, while the center frequency is denoted by $f_{c}$. Wavelet coefficients represent the correlation of a signal with wavelets at different scales. The squared modulus of the wavelet coefficients represents the distribution of the signal energy over time and reflects the localized energy intensity of the signal. Especially in the case of signals passing through damaged regions, the presence of damage in the region to be detected causes sudden fluctuations in the signal, which leads to changes in the local amplitude. This is reflected in the squared modulus sequence as a clear peak in the sequence. Therefore, we adopt the coefficient squared modulus obtained by CWT as the energy density signal. The squared modulus of the wavelet coefficients is defined as shown below:

(3) $$|C(a,b){|}^{2}={\left|\int_{-\infty }^{+\infty }f(t)\psi^{*}\left(\frac{t-b}{a}\right)dt\right|}^{2}$$

Before being fed into the encoders, each raw signal sequence x = [$x_{1}$, $x_{2}$, …, $x_{T}$] is normalized using min–max scaling to the range [0, 1]:

(4) $${\tilde{x}}_{t}=\frac{x_{t}-\min(x)}{\max(x)-\min(x)+\epsilon }$$

where $\epsilon =10^{-8}$ is a small constant employed to avoid division by zero. This ensures that all input signals are on a consistent scale, preventing features with larger numerical ranges from dominating the gradient updates during backpropagation.

### 2.2. Specific Encoders

#### 2.2.1. DS-Encoder

Due to the impact of noise and waveform distortion on the received data, alongside the instability of individual CNN units, enhancing the stability and robustness of the DS-encoder becomes crucial. To achieve this, we have designed a CNN block with residual connections, specifically tailored to extract features from damage signals $\left(f_{D_{i}}\in (1,13108)\right)$ within any set of filtered multimodal signals (where the subscript ii denotes the index of the actuator–sensor pair, and the segment borders refer to the time indices delimiting the direct wave arrival and boundary reflections). This approach effectively addresses the challenges of overfitting and gradient vanishing that may arise during the training process [21]. Furthermore, the incorporation of a batch normalization layer is intended to maintain consistency across the data. Ultimately, the features are compressed and output as a final feature vector $\left(\tilde{f_{D_{i}}}\right)$ through a one-dimensional max-pooling layer. The sequences of the convolutional and pooling operations are detailed below.

(5) $$\tilde{X_{l^{\prime }\times w^{\prime }}^{\left(m+1\right)}}=F\left(W_{l\times w}^{\left(m\right)}\cdot X_{t\times w}^{\left(m\right)}+B^{\left(m\right)}\right)$$

(6) $$P_{l^{\prime \prime }\times w^{\prime \prime }}^{\left(n\right)}=\underset{(i-1)K+1\leq t^{\prime }\leq \mathrm{iK}}{\max}\left\{X_{l^{\prime \prime }\times w^{\prime \prime }}^{\left(m+2\right)}\right\}$$

where m is the mth convolutional layer; $W_{l\times w}^{\left(m\right)}$ denotes the weight of the mth convolutional kernel; l is the length of the 1-D convolutional kernel; w denotes the width of the 1-D convolutional kernel and the input vector; $X_{t\times w}^{\left(m\right)}$ denotes the input vector; t is the length of the input vector; $\tilde{X_{l^{\prime }\times w^{\prime }}^{\left(m+1\right)}}$ denotes the output of the convolutional operation after one layer, which also serves as the input to the next convolutional layer; $l^{\prime }$ is the length of the output vector; $w^{\prime }$ denotes the width of the output vector; and F(·) denotes the ReLU activation function. In the max-pooling operation, $P_{l^{\prime \prime }\times w^{\prime \prime }}^{\left(n\right)}$ denotes the output after the max-pooling layer operation, n is the nth layer of the max-pooling layer; and $l^{\prime \prime }$ is the length of the final feature vector. Since the one-dimensional pooling layer does not alter the width of the vector, it maintains a width of $w^{\prime }$.

#### 2.2.2. SW-Encoder

**Figure 2:**
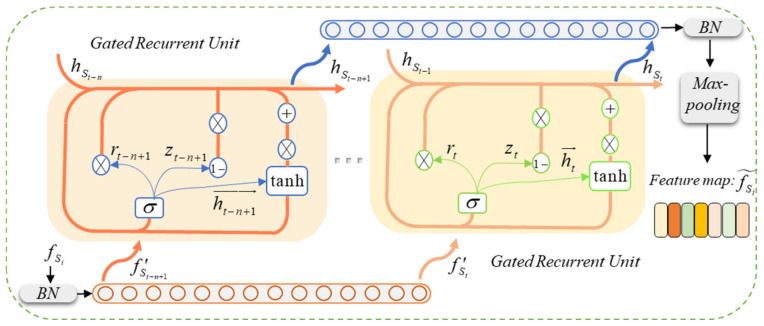
A schematic diagram of the SW-encoder.

The scattered wave signals derived from the baseline subtraction method are high-dimensional in terms of temporal length, and contain richer, more densely distributed feature information than raw damage signals [22,23]. The GRU is suitable for capturing the relationship between sequence data, has a strong long-range modeling capability, and is suitable for dense feature extraction [24]. Therefore, we use GRUs to construct the SW-encoder. In order to unify the structure of the specific encoders, we also design two batch normalization layers to keep the consistency of the data, and use GRUs to extract the features of the scattered wave signals. Figure 2 shows a flow diagram of the SW-encoder.

For a scattered wave signal $\left(f_{S_{i}}\in (1,13108)\right)$ in any signal group, the operation of the GRU at time t is given as follows:

(7) $$r_{t}=\sigma \left(W_{r}f_{S_{t}}^{\prime }+U_{r}h_{S_{t-1}}+b_{r}\right)$$

(8) $$z_{t}=\sigma \left(W_{z}f_{S_{t}}^{\prime }+U_{z}h_{S_{t-1}}+b_{z}\right)$$

(9) $$\vec{h_{S_{t}}}=\tanh\left(Wf_{S_{t}}^{\prime }+Ur_{t}h_{S_{t-1}}+b\right)$$

(10) $$h_{S_{t}}=\left(1-z_{t}\right)\vec{h_{S_{t}}}+z_{t}h_{S_{t-1}}$$

$r_{t}$ and $z_{t}$ denote the reset and update gates at time t, σ(·) denotes the sigmoid function; $h_{S_{t-1}}$ denotes the output of the previous moment, $\vec{h_{S_{t}}}$ denotes the hidden layer state at time t, $f_{S_{t}}^{\prime }$ denotes the output of the scattered wave signal $f_{S_{i}}$ after batch normalization, and W and U represent the weight matrix.

#### 2.2.3. ES-Encoder

**Figure 3:**
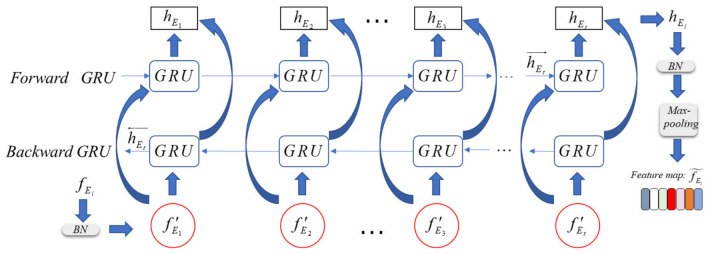
A schematic diagram of the ES-encoder.

When a signal traverses a region affected by damage, its characteristics undergo significant alterations, including distinct vibration modes before and after the damage [25], as well as variations in signal propagation speed [7]. The energy density signal accentuates more pronounced peaks when reflecting these changes. In this context, BiGRUs are particularly adept at adaptively learning diverse dynamic patterns from sequential data through their capacity for bidirectional information capture. Numerous studies [26,27,28] have demonstrated the superior modeling capabilities of BiGRUs for sequence data, which is why we have chosen to employ a BiGRU in the construction of the ES-encoder. A schematic representation of the ES-encoder’s structure is illustrated in Figure 3. The BiGRU is composed of two groups of GRUs that operate in opposing directions, allowing for feature extraction from the input sequence data in both forward and reverse orientations. As illustrated in Figure 3, for any given group of energy density signals, after undergoing batch normalization, the signal can be represented as $f_{E_{i}}^{\prime }=\left[f_{E_{1}}^{\prime },\;f_{E_{2}}^{\prime },f_{E_{3}}^{\prime },\ldots ,f_{E_{t}}^{\prime }\right]$. The hidden layer state is denoted as $\vec{h_{E_{t-1}}}$ at time t−1, with the input $f_{E_{t}}^{\prime }$. It is defined as follows:

(11) $$\vec{h_{E_{t}}}=Z\left(f_{E_{t}}^{\prime },\vec{h_{E_{t-1}}}\right)$$

where Z(·) represents the nonlinear transformation of the input variables. Similarly, the backward hidden layer state $\vec{h_{E_{t}}}$ is defined as follows:

(12) $$\overset{\leftarrow }{h_{E_{t}}}=Z\left(f_{E_{t}}^{\prime },\overset{\leftarrow }{h_{E_{t-1}}}\right)$$

(13) $$h_{E_{t}}=w_{t}\vec{h_{E_{t}}}+v_{t}\overset{\leftarrow }{h_{E_{t}}}+b_{t}$$

Here, $w_{t}$ and $v_{t}$ denote the forward hidden layer state weights and backward hidden layer state weights, respectively, and $b_{t}$ denotes the bias parameter.

### 2.3. Feature Fusion Strategy

**Figure 4:**
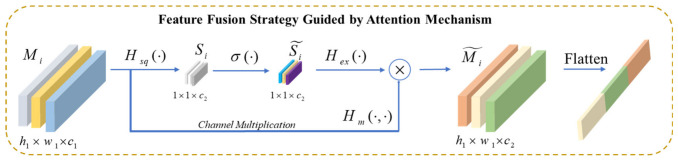
Flowchart of feature fusion guided by attention mechanisms.

To efficiently fuse the features obtained from different modalities, we employ a feature fusion strategy guided by the attention mechanism. The specific operational process is illustrated in Figure 4. Once the signals from different modalities are encoded, the resulting feature map $\left(M_{i}\in \left(f_{D_{i}},f_{S_{i}},f_{E_{i}}\right)\right)$ encompasses the features from all modalities. Through processes such as squeezing $\left(H_{sq}\left(\cdot \right)\right)$, excitation ${(H}_{ex}(\cdot ))$, and channel-wise multiplication ${(H}_{m}(\cdot ,\cdot ))$, the initial feature map $M_{i}\in \left(f_{D_{i}},f_{S_{i}},f_{E_{i}}\right)$ is transformed into a new feature map ${\tilde{M}}_{t}\in \left({\tilde{f}}_{D_{t}}^{\prime },{\tilde{f}}_{S_{t}}^{\prime },{\tilde{f}}_{E_{t}}^{\prime }\right)$ that captures more comprehensive global damage feature information, as represented in the following equations:

(14) $$H_{SE}=\sigma \left(W_{2}\delta \left(W_{1}M_{i}\right)\right)$$

(15) $$M_{i}=H_{m}\left(M_{i},S_{i}\right)$$

where δ, σ denote the ReLU activation function and sigmoid activation function, respectively, and $W_{1}$, $W_{2}$ denote the weight matrices of the two continuous linear layers.

### 2.4. Decoder and Implementation Details

After the feature fusion process, $\tilde{M_{i}}$ is decoded through three consecutive linear layers, which include Dropout layers to prevent overfitting during training. The final output prediction is generated through a softmax layer. The specific process is outlined as follows:

(16) $$y_{i}=Contact\left({\tilde{M}}_{i}\in \left(\tilde{f_{D_{i}}},\tilde{f_{S_{i}}},\tilde{f_{E_{i}}}\right)\right)$$

(17) $$y^{\prime \prime \prime }=Linear(y_{i})$$

(18) $$softmax\left(y_{i}^{\prime }\right)=\frac{e^{y_{i}}}{\sum_{i=1}^{n}e^{y_{i}}}$$

where $y_{i}$ denotes the output after the flattening operation. $y_{i}^{\prime \prime }$ is the output result after the linear layer decoding. Following the division of the set damage locations into zones, as described in the next section (Experimental Setup), the cross-entropy loss function is used, which is defined as follows:

(19) $$H(p,q)=-\sum_{i=1}^{n}p\left(x_{i}\right)\log q\left(x_{i}\right)$$

We adopt the cross-entropy loss for this 7-class classification task. Let p(x) denote the one-hot encoded ground-truth label vector and q(x) the predicted probability distribution from the softmax layer. The loss penalizes the deviation between the predicted and true label distributions. Accuracy is used as the evaluation metric, which measures the proportion of correct predictions. The accuracy is defined as follows:

(20) $$AR=\frac{T}{N}$$

T denotes the number of correct predictions, N is the total number of samples, and AR represents the accuracy rate.

To ensure the fairness of the experimental results, the same hyperparameters will be consistently applied throughout the training process. The specific hyperparameters are detailed in Table 1. The detailed parameters of the MSFN model architecture are shown in Table 2. The effectiveness of selecting these specialized encoders will be validated individually in the subsequent ablation experiments.

## 3. Experimental Setup

In this section, we utilize a comprehensive dataset obtained from the online platform for ultrasonic guided wave measurements [29], conducted under constant ambient conditions of 23 °C and 50% relative humidity. To ensure reproducibility and avoid data leakage, the full dataset (1914 samples) was randomly partitioned into 70% for training (1340 samples), 10% for validation (191 samples), and 20% for testing (383 samples). Stratified sampling was applied to maintain the class distribution across all subsets, ensuring that each of the seven damage zones is equally represented. The random seed was fixed at 42 to allow exact replication. The tested structure comprises a quasi-isotropic CFRP plate, specifically featuring a layup configuration of ([45/0/−45/90/−45/0/45/90] s) with dimensions of 500 × 500 × 2 mm.

To simulate damage on this plate, aluminum disks with a diameter of 10 mm were employed, and attached to the structure using adhesive tape, thereby representing reversible damage. The white circles in the diagrams in [30] indicated four closely spaced damage locations. Previous work in the literature employed adhesive patches to absorb some of the energy from the transmitted signal, thereby simulating damage and confirming the practical applicability of this approach [31]. In each measurement, the damage location was varied by individually adjusting one reversible damage element. It should be noted that adhesive-taped aluminum disks serve as a reversible and controllable proxy for damage, which allows systematic data collection under consistent conditions. However, they do not fully replicate the non-linear scattering and permanent nature of real fatigue cracks or delaminations.

**Figure 5:**
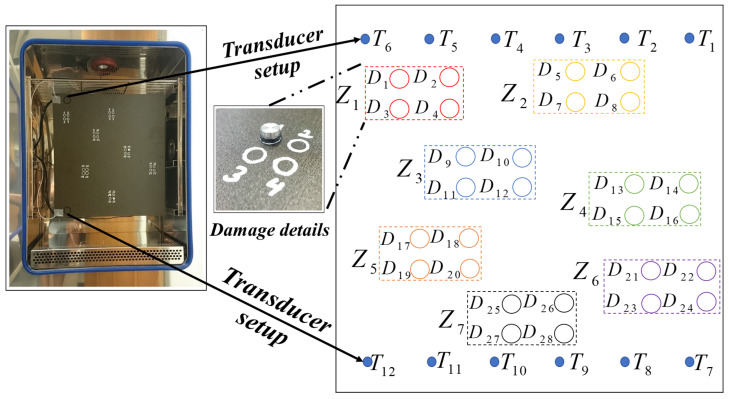
Detailed setup of the experiment.

Figure 5 illustrates the details of the damage and sensor setup as described in [30]. The sensors were arranged in two parallel rows from right to left. The excitation sequence began from one side and proceeded through to the other. The excitation signal employed in [30] was a 5-cycle Hann-filtered sine wave with center frequencies of 40 kHz, 60 kHz, 80 kHz, 100 kHz, and 120 kHz, as required by the subsequent experimental stages. In each measurement at a single center frequency, a total of 66 data channels were generated for damage localization, including 36 transmission channels for array cross-side transmission and 30 transmission channels for array same-side transmission, both containing feature information regarding the damage location. During each cyclic excitation, the reversible damage model was implemented by varying the damage locations with different placements on the CFRP plate, resulting in distinct damage instances. The damage locations were divided into 7 zones. In this study, data collected from receiver channels during each cycle at a single center frequency serves as the dataset, totaling 1914 data points. This dataset includes damage signals and baseline signals collected under similar temperature and humidity conditions.

**Figure 6:**
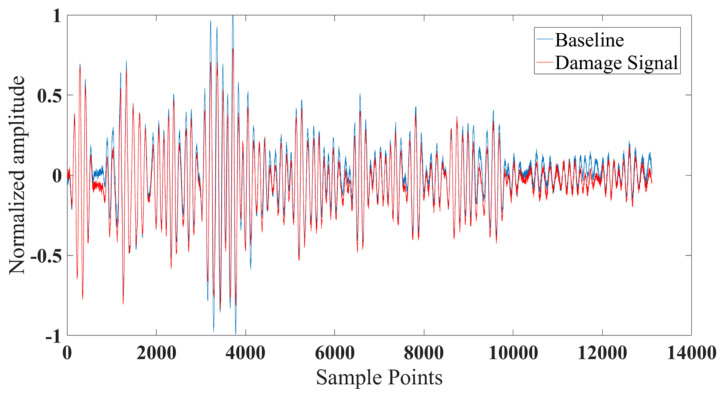
Raw damage signals and baseline signals received by $T_{6}-T_{12}$ with $D_{1}$ damage at a center frequency of 80 kHz.

To ensure uniform distribution across data classes in the data space, we allocate 420 data points as the test set. The test set consists of seven classes, corresponding to the seven damage zones, with each class containing over 60 data points. Figure 6 below presents the signals received by the actuator–receiver pair following the damage, at an excitation frequency of 80 kHz. In this figure, the blue signals represent the baseline signals, while the red signals denote the damage signals.

## 4. Results and Discussion

In this section, all experiments were conducted under the same environmental conditions. The experimental training environment was configured with the Windows 10 operating system, an Intel(R) Core(TM) i5-10500 CPU, and a GeForce RTX 2060 graphics processor (GPU) made in Chongqing. The deep learning models were developed using the Pytorch framework (2.12.0).

To mitigate the risk of overfitting given the limited experimental dataset (1914 samples), we employ several regularization strategies: (1) dropout layers with a rate of 0.3 are inserted in the decoder’s linear layers; (2) batch normalization is applied after each convolutional and recurrent layer to stabilize training; (3) early stopping with a patience of 30 epochs is used to halt training when the validation loss ceases to decrease.

### 4.1. Ablation Experiment

#### 4.1.1. Effectiveness of Multimodal Input Mode

To demonstrate the effectiveness of the multimodal input mode for damage localization in this experiment, we utilized three input signals: $f_{D}$ represents the damage timing signal, $f_{S}$ denotes the scattered wave signal, and $f_{E}$ corresponds to the energy density signal obtained from the scattered wave after CWT. We conducted comparative experiments using the following combinations: $\left(f_{D},f_{S}\right)$, $\left(f_{D},f_{E}\right)$, $\left(f_{S},f_{E}\right)$ and $\left(f_{D},f_{S},f_{E}\right)$. In order to ensure the fairness of the experiments, we uniformly use a dataset with a center frequency of 80 kHz for model training and testing. The experimental results presented in Table 3 indicate that our proposed multimodal input mode $\left(f_{D},f_{S},f_{E}\right)$ yields superior results, achieving a prediction accuracy of 99.04% for damage localization. In other input modes, accuracy rates show varying degrees of decline compared to the method proposed in this study. Notably, for the second input mode ($f_{D}$,$f_{E}$) and the third input mode ($f_{S}$,$f_{E}$), the experimental results are 95.95% and 96.19%, respectively.

**Figure 7:**
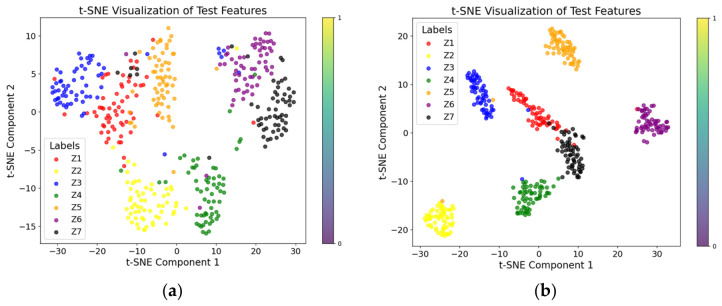

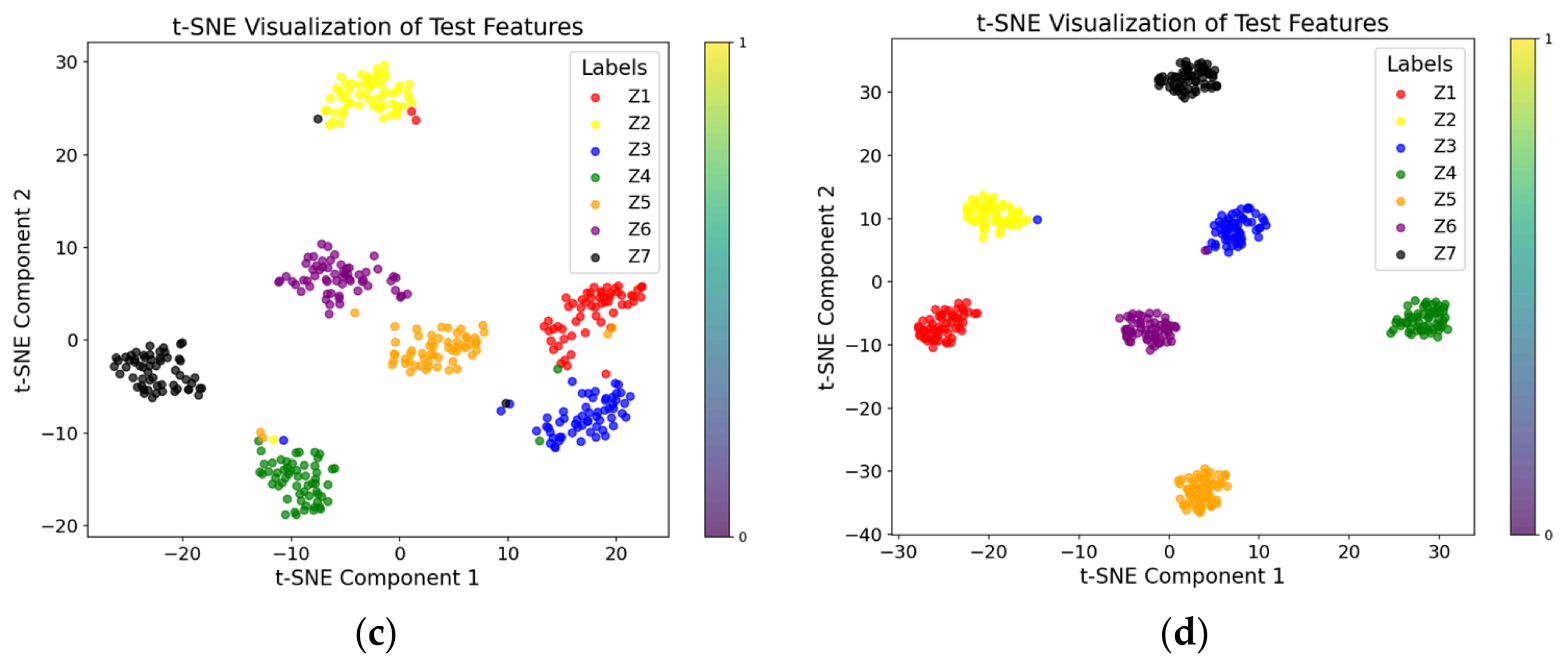
t-SNE visualization in different input modes: (**a**) $\left(f_{D},f_{S}\right)$, (**b**) $\left(f_{D},f_{E}\right)$, (**c**) $\left(f_{S},f_{E}\right)$, (**d**) $\left(f_{D},f_{S},f_{E}\right)$.

The accuracy of the second input mode is slightly lower than that of the third one. However, the damage signal in plate structure detection contains less damage feature information compared to the scattered wave signal, as the scattered wave signal is obtained through baseline subtraction, where the baseline signal contains information about the intact plate structure. Despite the reduced damage feature information in both modes, the results remain relatively close. Thus, we can reasonably conclude that the energy density signal contains richer and more diverse damage feature information than both the damage signal and the scattered wave signal. To further illustrate the results of the multimodal input mode, we employed t-distributed Stochastic Neighbor Embedding (t-SNE) for visualizing the classification outcomes [32]. From the visualization plots in Figure 7, it can be seen that when the input mode is a multimodal input mode, each type of damage is better distinguished and the prediction results have high accuracy. The prediction results in other input modes are slightly inferior. This also proves that our proposed multimodal input mode is reasonable and effective.

#### 4.1.2. Effectiveness of Specific Encoders and Attention-Guided Fusion Strategy

To demonstrate the efficacy of the proposed specific encoders and attention-guided fusion strategy in extracting and fusing diverse features within a multimodal input mode, we organized the experiments into two groups. Each group employed a distinct fusion strategy and different encoder architectures. For an objective evaluation of the experimental results, the model was trained and tested using a dataset with a center frequency of 80 kHz. Additionally, random Gaussian noise with a consistent distribution was incorporated into the test set.

**Figure 8:**
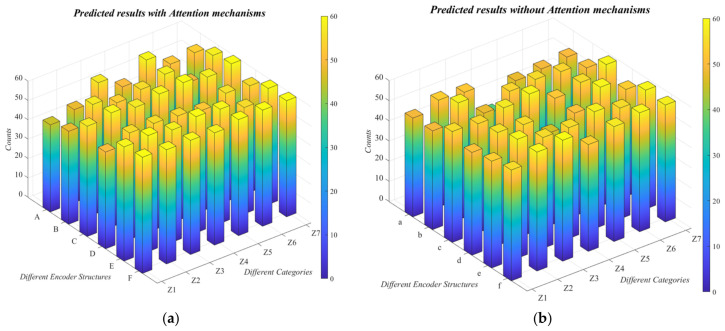
Damage localization results with different feature fusion strategies: (**a**) the strategy with an attention mechanism; (**b**) the strategy without an attention mechanism.

The experimental results presented in Table 4 indicate that when employing the same encoder structure alongside different feature fusion strategies, there are significant discrepancies in experimental accuracy. The attention mechanism-guided fusion strategy effectively aggregates various features, resulting in a feature map enriched with global damage feature information. Notably, this attention-guided fusion strategy does not substantially increase computational time and enhances model performance by 3.52%. Conversely, the conventional fusion strategy fails to adequately assign weights among different features, preventing the accentuation of discriminative features and thereby limiting the network’s ability to accurately characterize damage locations. To elucidate these findings further, Figure 8a,b provide visualizations of the damage localization results for each category outlined in Table 4. Here, A~F and a~f denote distinct encoder structures. $Z_{1}\sim Z_{7}$ represent various damage locations. The Z-axis illustrates the number of predicted results for each category of damage location, with an expected count of 60 for each category. A comparison of Figure 8a,b clearly demonstrates that the predicted damage outcomes generated by the attention mechanism-guided fusion strategy are generally more accurate.

To validate the effectiveness of the proposed specific encoders, we utilized the same training hyperparameters and dataset to evaluate networks with different encoder architectures. Initially, we designated the CNN block with residual connections (CNN + ResCon), the GRU and the BiGRU as encoders for the three signals, respectively. As indicated by the first three rows of Table 4, the accuracy remains generally low, even with the attention mechanism employed in the fusion strategy. Notably, while the accuracy can reach 97.99% when all three encoders use the BiGRU, the training time surges to 2545.49 s, highlighting the extensive number of parameters associated with the BiGRU. Consequently, in the subsequent validation phase, we selected the CNN + ResCon module, which incurs a lower time overhead, as the DS-Encoder. The experimental results presented later in Table 4 show a marked reduction in computational burden when the network integrates the CNN + ResCon module. To balance the dual objectives of accuracy and computational time, we designated the BiGRU module as the ES-encoder and the GRU module as the SW-encoder to enhance accuracy. The subsequent results reveal that the accuracy of the experiment is as high as 98.97% and 95.47% after we have efficiently combined the three modules. This improvement stems from our previous findings, which demonstrate that the energy signal contains richer time–frequency information, and that the BiGRU can effectively capture temporal correlations within these features, thereby enhancing damage detection accuracy.

### 4.2. Robustness and Generalizability Testing

#### 4.2.1. Comparison of Robustness Among Different Networks

**Figure 9:**
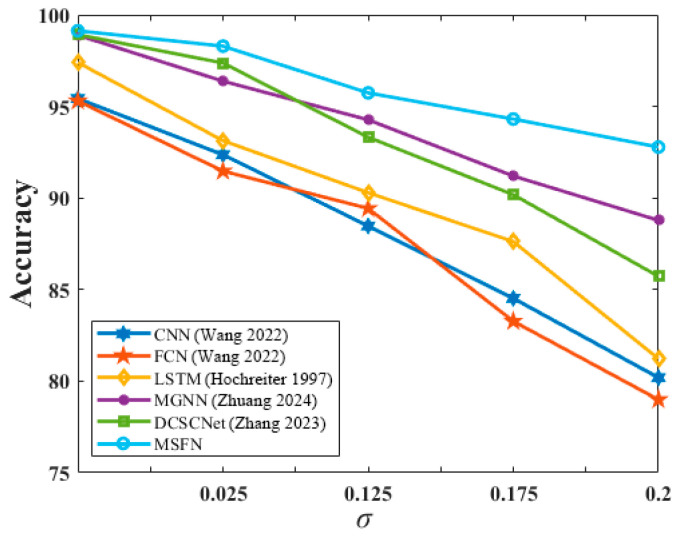
Comparison between different networks [13,19,21,33].

In typical damage detection processes, the acquired signals generally contain clutter and noise. To validate the interference resistance of the MSFN, we systematically introduced five different sets of Gaussian noise into the test set to assess the noise resilience of various models under different conditions. Figure 9 illustrates the testing accuracy under different noise conditions. When the noise influence is minimal, most models perform quite well, with the MSFN, DCSCNet [21], and MGNN [19] models achieving accuracies around 99%. However, in real environments, the impact of noise cannot be overlooked. When the effects of noise and clutter suddenly increase, the predictive localization results of most models inevitably deteriorate. Methods such as CNNs [13], FCNs [13], LSTM [33], and DCSCNet [21], which utilize a single-modal input approach, struggle to extract multiple distinct feature types, resulting in a drop in accuracy to approximately 80%. Thus, these models exhibit relatively poor robustness. In addition, the MGNN [19] has different multimodal input modes. The MGNN [19] only fuses the damage signals received from the sensors and the corresponding CWT-transformed signals. In contrast, the MSFN has features of different modalities extracted under multiple signal fusion, which makes the interference caused by noise have a limited effect on the feature extraction process. Therefore, the test results under harsh noise conditions are better. When σ=0.4, the accuracy of our method can still reach 92.78%, while the MGNN can only reach 88.78% accuracy in this case. This is due to the fact that our method adopts a better attention mechanism as the feature fusion strategy as well as a more comprehensive multimodal input mode, which constructs a more complex and comprehensive nonlinear mapping between damage features and damage locations.

#### 4.2.2. Validation of MSFN’s Generalization Under Different Feature Distributions

**Figure 10:**
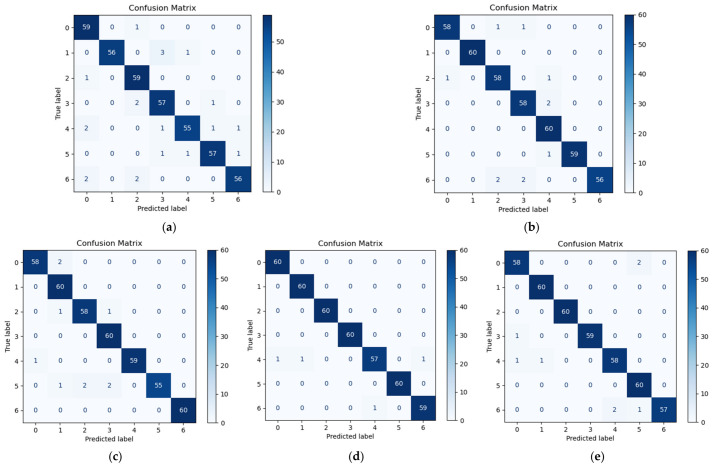
Experimental results with different feature distributions: (**a**) f=40 kHz, (**b**) f=60 kHz, (**c**) f=80 kHz, (**d**) f=100 kHz, (**e**) f=120 kHz.

To assess the generalization capability of the MSFN model, random Gaussian white noise with the same distribution was added to the filtered signals to evaluate the model’s performance and effectiveness under noise influence. A total of five different excitation frequencies were utilized to examine the model’s multimodal feature perception and fusion capabilities across different center frequency signals. This approach allows us to test the model’s ability to perceive and extract features from varying distributions. The resulting visualized confusion matrix is presented in Figure 10. From the results presented in Table 5, it can be concluded that our proposed method exhibits strong feature sensing capabilities even when faced with different feature distributions under the same noise conditions, achieving an accuracy of approximately 97%. This sufficiently demonstrates the model’s robust generalization ability. From the results presented in Figure 10a, it is noteworthy that when the center frequency is equal to 40 kHz, the experimental accuracy experiences a decline. This reduction is attributed to the signal preprocessing, where a high-pass filter with a cutoff frequency of 20 kHz was applied to eliminate noise, resulting in some loss of low-frequency damage feature information. In contrast, at 100 kHz, the accuracy can reach as high as 99.05%. The experimental results indicate that the MSFN exhibits a robust capability in characterizing damage locations when the damage feature information is comprehensive. It can perform accurate and rapid damage localization, demonstrating strong adaptability to signals with diverse feature distributions. This evidence confirms the model’s exceptional generalization ability.

## 5. Conclusions

In this paper, we propose a novel method for damage localization using a Multimodal Feature Sensing and Fusion Neural Network (MSFN) based on UGWs. This method primarily addresses the challenges associated with existing deep learning models that rely on UGWs, specifically the issues of single-feature modality, poor robustness, and low localization accuracy. Firstly, the proposed approach employs an innovative multimodal input mode that integrates different types of signals into the network. By combining time-domain and energy information, this mode enhances the diversity of features and improves the network’s resilience to noise. Secondly, to facilitate accurate and efficient feature extraction, the MSFN incorporates specific encoders tailored for different signal types. Furthermore, the network utilizes an attention mechanism to guide the feature fusion strategy, allowing for the adaptive aggregation of diverse features, which further augments the network’s capacity to accurately represent damage locations. We validated the effectiveness of the proposed method using a public dataset available on an online platform for ultrasonic guided wave measurements. Through a series of ablation experiments, we demonstrated the efficacy of the multimodal input mode, specific encoders, and the attention mechanism-guided fusion strategy. In robustness testing, the MSFN achieved a localization accuracy of 98.13% under noisy conditions. Additionally, in evaluating generalizability, the accuracy of the MSFN in damage localization remained at approximately 97.85% when processing signals with varying feature distributions. The high accuracy, strong noise resilience, and excellent generalization of our method significantly outperform existing deep learning approaches.

Despite these achievements, several limitations must be acknowledged, which we present here along a coherent pathway from data to deployment. First, the use of adhesive-taped aluminum disks as damage proxies oversimplifies real damage such as delamination and fatigue cracks, which exhibit permanent, non-linear scattering not replicable by tape. We are currently planning validation on CFRP specimens with impact-induced delamination and fatigue cracks. Second, framing localization as a 7-class classification inherently limits spatial resolution; a natural extension is to reframe the decoder as a regression network for continuous (x, y) coordinate outputs, which we will pursue once datasets with finer spatial annotations become available. Third, environmental factors including ambient temperature fluctuations, varying clamp pressures, and CFRP anisotropy can alter wave velocity, amplitude, and dispersion. Our time-shift experiments provide preliminary insights, but practical deployment will require baseline update strategies, temperature compensation, or material-specific fine-tuning. Fourth, the current model is evaluated on damage within the sensor grid; out-of-grid or multi-damage scenarios may degrade performance, necessitating future augmentation with such configurations. Fifth, deployment on resource-constrained edge hardware requires further optimization to meet real-time requirements. Regarding the latter two points, we emphasize that the proposed framework is extensible: the network can be retrained on augmented data covering out-of-grid geometries, and the attention-guided fusion module is lightweight enough to benefit from quantization and pruning for edge deployment.

To situate these future developments within a structured engineering framework, we map our methodology onto the CRISP-DM lifecycle, which has been successfully adapted for NDT research. The Data Understanding and Data Preparation phases correspond to our signal preprocessing and CWT-based feature extraction; the Modeling phase corresponds to the MSFN architecture; and the Evaluation phase corresponds to our robustness and generalization tests. The Deployment phase, which covers model compression, on-target hardware validation, integration with data-acquisition systems, and automated sensor array layout optimization, constitutes our primary future direction. This lifecycle perspective, together with the structured roadmap above, positions the MSFN as a robust foundation for intelligent SHM with a clear path toward practical engineering translation.

## Figures and Tables

**Table 1:** The hyperparameters of the training.

Hyperparameters	Value
Learning rate	1 × 10^−4^
Batch size	16
Epoch	250
Optimizer	SGD

**Table 2:** The detailed structure of the MSFN.

Component	Layer	Input Dim	Output Dim
DS-Encoder	1D-CNN, BN, ReLU	1 × 13,108	8 × 6553
	1D-CNN, BN, ReLU	8 × 6553	1 × 3275
	Max-Pooling	1 × 3275	1 × 1637
SW-Encoder	BN, GRU, BN	1 × 13,108	1 × 3275
	Max-Pooling	1 × 3275	1 × 1637
ES-Encoder	BN, BIGRU, BN	1 × 13,108	1 × 3275
	Max-Pooling	1 × 3275	1 × 1637
Feature Fusion	Attention, Flatten	3 × 1637	1 × 4911
Decoder	Linear-1	4911	128
	Linear-2	128	32
	Linear-3	32	7

**Table 3:** The accuracy of different input modes.

Different Input Modes	Accuracy (%)
$\left(f_{D},f_{S}\right)$	89.53%
$\left(f_{D},f_{E}\right)$	95.95%
$\left(f_{S},f_{E}\right)$	96.19%
$\left(f_{D},f_{S},f_{E}\right)$	99.04%

**Table 4:** The detailed experimental results.

Encoder Structure Designator	Feature Fusion Strategy	DS-Encoder	SW-Encoder	ES-Encoder	Testing Loss	Training Time (s)	Accuracy (%)
*A*	With attention mechanisms	CNN + ResCon			0.8792	322.09	85.71%
*B*		GRU			0.3243	2007.42	89.99%
*C*		BiGRU			0.0833	2545.49	97.99%
*D*		CNN + ResCon	GRU	GRU	0.3352	1426.83	90.23%
*E*		CNN + ResCon	BiGRU	BiGRU	0.0920	1814.24	97.92%
*F*		CNN + ResCon	GRU	BiGRU	0.0574	1655.19	98.97%
*a*	Without attention mechanisms	CNN + ResCon			1.5386	312.00	80.47%
*b*		GRU			0.5153	1970.80	87.33%
*c*		BiGRU			0.2051	2572.80	92.19%
*d*		CNN + ResCon	GRU	GRU	0.3646	1476.83	88.33%
*e*		CNN + ResCon	BiGRU	BiGRU	0.2573	1849.54	93.80%
*f*		CNN + ResCon	GRU	BiGRU	0.1924	1599.73	95.47%

**Table 5:** The accuracy of different center frequencies.

Center Frequencies (kHz)	Accuracy (%)
40	95.71%
60	97.38%
80	97.85%
100	99.05%
120	98.09%

## Data Availability

The data that support the findings of this study are available from the corresponding authors upon reasonable request.
